# The relationship between depressive syndrome and suicidal risk in patients with acute schizophrenia

**DOI:** 10.3325/cmj.2013.54.436

**Published:** 2013-10

**Authors:** Dario Bagarić, Petrana Brečić, Draženka Ostojić, Vlado Jukić, Ana Goleš

**Affiliations:** 1 Department for Integral Psychiatry, Psychiatric University Hospital Vrapče, Zagreb, Croatia; 2 Department for Treatment and Rehabilitation, Psychiatric University Hospital Vrapče, Zagreb, Croatia; 3 Department for Diagnostics and Intensive Care, Psychiatric University Hospital Vrapče, Zagreb, Croatia; 4 Department for Forensic Psychiatry, Psychiatric University Hospital Vrapče, Zagreb, Croatia; 5 Psychiatric University Hospital Vrapče, Zagreb, Croatia

## Abstract

**Aim:**

To determine the relationship between scores on five factors of the Positive and Negative Syndrome Scale (PANSS) and Calgary Depression scale for Schizophrenia (CDSS) and scores on the InterSePT Scale for Suicidal Thinking (ISST) in patients with acute schizophrenia.

**Methods:**

Data were collected on sociodemographic and clinical characteristics of 180 drug-treated in-patients with acute schizophrenia. Their symptoms were assessed with PANSS, CDSS, and ISST and correlations between the scores were calculated. Statistically significant correlations were included in the logistic regression analysis to identify predictors of suicidal risk.

**Results:**

CDSS (*P* < 0.001) score and negative (*P* < 0.001), disorganized (*P* = 0.041), emotional (*P* < 0.001), and total score on PANSS (*P* < 0.001) showed a significant positive correlation with ISST. Stepwise logistic regression analysis revealed that CDSS scores (odds ratio [OR] 5.18; confidence interval [CI] 1.58-16.95), and disorganized (0.90; 0.81-0.99) and emotional (1.15; 1.01-1.30) factors of PANSS were predictors of suicidal risk.

**Conclusion:**

Our results suggested a considerable association between depressive syndrome as assessed by the PANSS emotional factor and CDSS score and suicidal risk in patients with acute schizophrenia.

Suicide is a major cause of death in patients with schizophrenia. The estimated lifetime rate for suicide in schizophrenia is about 5% (1). Many studies explored the relationship between psychiatric symptomatology and suicide in patients with schizophrenia and obtained contradictory results. However, most of the studies showed a strong association between suicide and depressive symptoms, especially in the acute phase of the illness (2-9). Among hospitalized patients, suicidal risk peaks shortly after admission and shortly after discharge from hospital (10). The association between positive and negative symptoms and suicidal risk is less clear. A large meta-analysis showed inconsistent results on the role of positive and negative symptoms in suicidal risk in patients with schizophrenia (11). Schizophrenia has been considered a clinically heterogeneous entity and – according to contemporary concepts – a syndrome with diverse symptoms (12). One of the major attempts to make this heterogeneous entity more structured was Positive and Negative Syndrome Scale (PANSS), which consists of three symptom clusters: positive, negative, and general psychopathology (13). It is still the most frequently used instrument for rating schizophrenia symptoms, which are currently clustered into five factors that are more appropriate for description of relevant heuristic clinical dimensions called “positive,” “negative,” “emotional,” “excitement,” and “disorganization” (14-23). Although a few models of universal item distribution have been proposed (24-27), the consensus is still lacking.

To help distinguish schizophrenia symptoms from depression and suicidal behavior, new questionnaires were developed. Calgary Depression Scale for Schizophrenia (CDSS) (28) was specifically developed to assess the level of depression in schizophrenia and found to be superior to other depression-rating scales (29,30). Lindenmayer et al (31) derived the InterSePT Scale for Suicidal Thinking (ISST) from the Scale for Suicide Ideation (32) by adjusting the scale for measuring the suicidal risk in depressed population and assessing current suicidal ideation in patients with schizophrenia and schizoaffective disorder (33-35).

The phenomenon of suicidal behavior still remains unexplored. Besides psychological, recent studies are focused on biological factors (36,37). Our study was designed to determine the relationship between scores on ISST, CDSS, and symptom clusters derived from five-factor PANSS analysis in a group of hospitalized drug-treated patients with acute schizophrenia.

## Methods

### Participants

This cross-sectional study included patients recruited from the Department for Diagnostics and Intensive Care and the Department for Treatment and Rehabilitation, Psychiatric University Hospital Vrapče between January 2007 and June 2009. Patients of both sexes, aged over 18 years, with intellectual and legal capacity to participate in the study, who were diagnosed with schizophrenia according to DSM-IV criteria (38) by two independent evaluators were eligible for the study. Exclusion criteria were treatment with antidepressants and any co-morbid psychiatric or somatic disorder that might influence clinical presentation, ie, alcohol or substance abuse, psychoorganic syndrome, neurological trauma or disease, or severe somatic disease.

The study sample consisted of 180 drug-treated inpatients with a mean (range) PANSS score of 98.0 (86.3-109.0), which is equivalent of “markedly ill” according to the Clinical Global Impression Scale (39) (Table 1).

**Table 1:** Demographic and clinical characteristics of patients with acute schizophrenia*

Variables	Men (n = 95)	Women (n = 85)	Total (n = 180)	*P*
Age (years, median and interquartile range)	29.0 (25.0-39.0)	36.0 (27.0-47.0)	32.0 (25.0-43.5)	0.002^‡^
Duration of illness (years, median and interquartile range)	2.0 (0.5-8.0)	3.0 (1.0-9.3)	2.9 (0.8-8.5)	0.272^‡^
Age of onset (years, median and interquartile range)	25.0 (21.0-29.9)	29.8 (23.3-38.2)	27.8 (21.9-33.3)	<0.001^‡^
No. of previous hospitalizations (median and interquartile range)	1.0 (0.0-3.0)	2.0 (0.0-4.0)	1.5 (0.0-4.0)	0.326^‡^
No. of previous suicide attempts (median and interquartile range)	0.0 (0.0-1.0)	0.0 (0.0-1.0)	0.0 (0.0-1.0)	0.405^‡^
Family history of (No., %):^†^				
schizophrenia	33 (34.7)	19 (22.4)	52.0 (28.9)	0.096^§^
depression	5 (5.3)	6 (7.1)	11.0 (6.1)	0.849^§^
suicide	9 (9.5)	14 (16.5)	23.0 (12.8)	0.238^§^
PANSS				
total score	102.0 (90.0-111.0)	93.0 (84.0-105.0)	98.0 (86.5-109.0)	0.001^‡^
positive (median and interquartile range)	27.0 (22.0-30.0)	25.0 (21.0-28.0)	26.0 (21.5-29.0)	0.012^‡^
negative (median and interquartile range)	26.0 (22.0-30.0)	24.0 (21.0-29.0)	26.0 (21.0-29.0)	0.146^‡^
disorganized (median and interquartile range)	33.0 (28.0-38.0)	30.0 (27.0-35.0)	31.0 (27.5-36.0)	0.003^‡^
excitement (median and interquartile range)	27.0 (24.0-31.0)	24.0 (21.0-29.0)	26.0 (22.0-30.00)	0.002^‡^
emotional (median and interquartile range)	28.0 (25.0-31.0)	27.0 (24.0-30.0)	27.0 (24.0-31.0)	0.113^‡^
CDSS total score (median and interquartile range)	6.0 (0.0-12.0)	5.0 (2.0-11.0)	5.0 (0.0-12.0)	0.892^‡^
ISST total score (median and interquartile range)	1.0 (0.0-6.0)	0.0 (0.0-2.0)	0.0 (0.0-4.0)	0.004^‡^

*PANSS – Positive and Negative Syndrome Scale; CDSS – Calgary Depression Scale for Schizophrenia; ISST – Intersept Scale for Suicidal Thinking.

^†^Some patients had multiple heredity.

^‡^Mann-Whitney test.

^§^χ^2^ test.

Participation in the study was voluntary. All patients gave informed consent. The study was approved by the Ethics Committees of the Psychiatric University Hospital Vrapče and the Osijek University School of Medicine.

### Clinical assessment

PANSS was used for evaluation of schizophrenia symptoms (13). Factors were calculated using five-factor model of PANSS by Van der Gaag et al (24,25) according to the formula:

1) Positive: delusions (P1) + hallucinatory behavior (P3) + unusual thought content (G9) + suspiciousness/persecution (P6) + grandiosity (P5) + somatic concerns (G1) + lack of judgment and insight (G12) + active social avoidance (G16) – difficulty in abstract thinking (N5)

2) Negative: lack of spontaneity and conversation flow (N6) + blunted affect (N1) + emotional withdrawal (N2) + passive/apathetic social withdrawal (N4) + motor retardation (G7) + poor rapport (N3) + active social avoidance (G16) + uncooperativeness (G8) + disturbance of volition (G13) – conceptual disorganization (P2)

3) Disorganization: stereotyped thinking (N7) + poor attention (G11) + disorientation (G10) + conceptual disorganization (P2) + difficulty in abstract thinking (N5) + mannerisms/posturing (G5) + lack of judgment and insight (G12) + disturbance of volition (G13) + preoccupation (G15) + unusual thought content (G9)

4) Excitement: poor impulse control (G14) + excitement (P4) + hostility (P7) + uncooperativeness (G8) + grandiosity (P5) + poor rapport (N3) + tension (G4) + active social avoidance (G16)

5) Emotional: anxiety (G2) + depression (G6) + guilt feelings (G3) + tension (G4) + suspiciousness/persecution (P6) + somatic concerns (G1) + preoccupation (G15) + active social avoidance (G16).

CDSS was used to evaluate depressive symptoms. To distinguish patients with depression from those without depression, the value of 7 was used as a cut-off point. The same value was used by Addington et al to distinguish between depressive patients and patients without depression (40), by Bressan et al to distinguish between patients with major depression and patients with moderate or without depression (41), and Müller et al to distinguish between patients with moderate and severe depression and those with mild depression or without depression (42).

ISST was used for the assessment of current suicidal ideation; the cut-off value of 6 was used to distinguish between patients with and without suicidal risk (43).

Median time from admission to psychometric evaluation was 4.0 days (25% quartile: 3.0 days, 75% quartile: 4.0 days). PANSS, CDSS, and ISST measurements were performed on the same day by three independent psychiatrists blinded to the scores on other scales.

### Statistical analysis

Normality of data distribution was assessed with Smirnov-Kolmogorov test. Since only PANSS showed a normal distribution, nonparametric tests were used in the analysis. Differences between male and female participants were analyzed with χ^2^ test (categorical values) and Mann-Whitney U test (quantitative values). Spearman correlation coefficients were calculated between ISST score and five PANSS factors and CDSS score. Logistic regression analysis was used to test the relationship between ISST scores (dichotomized as <6 and ≥6) as a dependent variable and sample variables (sex, suicide in family, schizophrenia in family, previous attempt of suicide), PANSS scores (negative, disorganization, emotional), and CDSS score (dichotomized as <7 and ≥7) as independent variables. A stepwise model was used. Sample variables were entered in the first block and scale scores in the second. *P*-values <0.05 were considered statistically significant. The analyses were performed using the SPSS for Windows, release 17 (SPSS Inc., Chicago, IL, USA).

## Results

Cronbach alpha coefficients showed high internal consistency for CDSS and ISST scales (αCDSS = 0.92 and αISST = 0.96). Internal consistencies of PANSS factors formed according to van der Gaag (24,25) were somewhat lower, but still high or within the acceptable range (α_positive_ = 0.73; α_negative_ = 0.83; α_disorganization_ = 0.81; α_excitement_ = 0.70; α_emotional_ = 0.64).

Men had higher ISST scores, PANSS total score, and positive, disorganized, and excitement PANSS factor scores than women (Table 1). Patients with heredity of suicide had higher ISST scores [C = 6.0 (Q1-Q3) = 1.0-11.0 vs 0.0 (0.0-3.0); *P* = 0.003; Mann-Whitney U test], as well as patients with heredity of schizophrenia [1.0 (0.0-6.5 vs 0.0 (0.0-3.0); *P* = 0.039]. Heredity of depression was not associated with higher ISST score. Patients with more suicide attempts had higher ISST scores (Table 2). CDSS, PANSS total score, and negative, disorganized, and emotional PANSS factor scores positively correlated with ISST scores (Table 2). CDSS also showed a strong correlation with emotional PANSS factor (rho = 0.508).

**Table 2:** Correlation between ISST scores and patient variables, CDSS and PANSS scores*

Variable	Correlation with ISST	*P^†^*
Age	0.019	0.803
Sex	-0.217	0.003
Heredity of depression	0.097	0.194
Heredity of schizophrenia	0.154	0.039
Heredity of suicide	0.221	0.003
Duration of illness	0.127	0.088
Age of onset	-0.058	0.438
No. of previous hospitalizations	0.040	0.592
No. of previous suicide attempts	0.445	<0.001
PANSS-positive	0.118	0.116
PANSS-negative	0.349	<0.001
PANSS-disorganized	0.153	0.041
PANSS-excitement	0.056	0.456
PANSS-emotional	0.569	<0.001
PANSS-total	0.313	<0.001
CDSS	0.728	<0.001

*ISST – Intersept Scale for Suicidal Thinking; CDSS – Calgary Depression Scale for Schizophrenia; PANSS – Positive and Negative Syndrome Scale.

^†^Spearman's rho.

Of 180 patients, 79 (43.9%) had CDSS score equal or higher than 7, and 34 (43.0%) had ISST score equal or higher than 6.

We performed a stepwise logistic regression analysis using all variables with moderate or high association with ISST as dependent variables. We entered patients' variables in the first block and added CDSS and PANSS factors correlating with ISST in the second block. Variables in the first block explained 61% of variance in the dependent variable, with previous attempt of suicide, sex (men had greater odds for higher ISST), and suicide in family as significant predictors. Adding PANSS and CDSS scale scores in the second block explained additional 10% of variance in the criterion. Previous attempt of suicide, male sex, and suicide in family remained significant with PANSS disorganized factor score, PANSS emotional factor score, and CDSS score as additional predictors (Table 3).

**Table 3:** Logistic regression model for Intersept Scale for Suicidal Thinking (ISST) scores*

	Predictors:	OR (95%CI)	Naglekerke R2
Block 1	sex	0.18 (0.11-0.29)^†^	0.61
	heredity of schizophrenia	1.00 (0.42-2.43)	
	heredity of suicide	4.31 (1.42-13.10)^†^	
	previous suicide attempt	11.84 (4.82-29.10)^†^	
Block 2	sex	0.15 (0.06-0.39)^†^	0.71
	heredity of schizophrenia	0.96 (0.35-2.65)	
	heredity of suicide	3.95 (1.11-14.06)^†^	
	previous attempt	10.92 (3.95-30.14)^†^	
	CDSS (<7 / ≥7)	5.18 (1.58-16.95)^†^	
	PANSS negative	0.96 (0.85-1.08)	
	PANSS disorganized	0.90 (0.81-0.99)^†^	
	PANSS emotional	1.15 (1.01-1.30)^†^	

*CDSS – Calgary Depression Scale for Schizophrenia; PANSS – Positive and Negative Syndrome Scale.

^†^Significant predictor (*P* < 0.05).

## Discussion

Our study showed that the PANSS total score, disorganized, negative, and emotional PANSS factor scores, and CDSS were positively correlated with suicidal risk. Among PANSS factors, emotional factor showed the strongest correlation. The CDSS showed the strongest correlation of all assessed variables, which was also found by Lindermayer et al (30). A significant positive correlation was also found between suicidal risk and male sex, heredity of schizophrenia, heredity of suicide, and number of previous suicide attempts.

The logistic regression model revealed that CDSS and emotional and disorganized PANSS factors were predictors of suicidal risk. Among patients’ variables, male sex, heredity of suicide, and previous suicidal attempts were predictors of suicidal risk. Similar findings were shown in previous studies (7,44-50).

The study was centered on associations between clinical features of acute schizophrenia and suicidal risk, which were assessed in a distinct constellation of psychometric tools and their interpretations. Contrary to other clusters of symptoms (with the exception of disorganized), the study proved depressive syndrome as a stable and strong predictor of suicidal risk, regardless of the related variable. Both CDSS and emotional factor, which is considered the PANSS equivalent of approximation of depressive syndrome (51), were predictors. Predictive value of disorganized cluster should be explored in further studies.

Results should be interpreted in the context of five-factor model. Although several universal models have been proposed, there is still no consensus. We chose the model by van der Gaag et al, which, unlike other item-restrictive universal models (26,27), uses multiple and negative item loadings and, therefore, is item-redundant. It is possible that other models would show different results with the same data. Furthermore, because causal or inductive associations between schizophrenia symptoms and PANSS items are still unknown, the question remains whether the selection of other items, not related to any of the suggested five-factor models, would show stronger associations. Especially interesting is the emotional factor, which showed the strongest correlation with suicidal risk. In Van der Gaag’s model, it consists of eight items (G2 + G6 + G3 + G4 + P6 + G1 + G15 + G16) and is highly different from equivalent factors of other models. For instance, it has twice as many items as emotional factor in the model by El Yazaji et al (G1 + G2 + G3 + G6) and has also shown a significant correlation with CDSS (51). One of PANSS disadvantages is multidimensionality – item scores and associated description of each item often describe different psychopathological constructs. Given that depression is one of the PANSS items, PANSS measures otherwise poorly differentiated depressive syndrome consisting of mood, vegetative, and cognitive symptoms. Additionally, there is no item that explicitly investigates suicidality. We found a positive correlation between negative symptoms and suicidal risk, contrary to some previous studies (7,52,53). This finding could be explained by the overlap between negative and depressive symptoms and difficulties in their differentiation with respect to poor conceptualization, which is the result of unknown nosology of each symptom and syndrome. Recognition of depressive symptoms should be an integral part of routine clinical evaluation of patients with schizophrenia. Some aspects of schizophrenia may mask depression symptoms and complicate their treatment. Since clinicians are usually focused on positive and negative symptoms of illness, depression often remains unrecognized and underestimated. Patients with predominantly positive symptoms, who respond well to antipsychotic therapy, are at the greatest risk of unrecognized depression. As negative symptoms are less correlated with increased risk of suicidal behavior, their overlap with depressive symptoms and unrecognized depression might lead to inadequate treatment and increased suicidal risk.

The limitation of our study was a relatively small study sample with a considerable proportion of depressive patients. Seventy-nine patients were depressive, 43% of whom were at increased suicidal risk. This high percentage of depressive patients in our sample could be explained with a high proportion of patients with first episode of schizophrenia, who are known to have a higher incidence of depressive symptoms than patients with multiple episodes of schizophrenia (54). A relatively small number of previous suicidal attempts was also the result of relatively high proportion of patients with first episode of schizophrenia, which might weaken the validity of related correlation.

In our study, antidepressant treatment was one of the exclusion criteria. Although antipsychotic genesis of depressive syndrome in schizophrenia is often mentioned, Krakowski et al (55) found that antipsychotics might have two-sided effect on depressive symptoms, while Siris et al (56) did not find any difference in the prevalence of depression in schizophrenia between patients who were and those who were not treated with antipsychotics. A study with drug-untreated patients might show different results.

In conclusion, in their everyday practice, clinicians are often focused on the most prominent aspect of clinical features in patients with acute schizophrenia, ie, on productive and disorganized symptoms, which may mask depressive symptoms. These symptoms were shown to be strongest predictors of suicidal risk and, therefore, should be carefully assessed. The same caution is required in assessing the overall patient history, clinical presentation, and heteroanamnestic data.
